# The University of Michigan Dioxin Exposure Study: Methods for an Environmental Exposure Study of Polychlorinated Dioxins, Furans, and Biphenyls

**DOI:** 10.1289/ehp.11777

**Published:** 2008-12-22

**Authors:** David H. Garabrant, Alfred Franzblau, James Lepkowski, Brenda W. Gillespie, Peter Adriaens, Avery Demond, Barbara Ward, Kathy LaDronka, Elizabeth Hedgeman, Kristine Knutson, Lynn Zwica, Kristen Olson, Timothy Towey, Qixuan Chen, Biling Hong

**Affiliations:** 1 Department of Environmental Health Sciences and Risk Science Center, University of Michigan School of Public Health, Ann Arbor, Michigan, USA;; 2 Survey Research Center, Institute for Social Research, University of Michigan, Ann Arbor, Michigan, USA;; 3 Department of Biostatistics, University of Michigan School of Public Health, Ann Arbor, Michigan, USA;; 4 Department of Civil and Environmental Engineering, University of Michigan College of Engineering, Ann Arbor, Michigan, USA;; 5 Survey Research and Methodology Program, University of Nebraska–Lincoln, Gallup Research Center, Lincoln, Nebraska, USA

**Keywords:** biomonitoring, diet, dioxins, environmental exposure, epidemiology, population-based, serum, soil, survey

## Abstract

**Background:**

The University of Michigan Dioxin Exposure Study (UMDES) was undertaken in response to concerns that the discharge of dioxin-like compounds from the Dow Chemical Company facilities in Midland, Michigan, resulted in contamination of soils in the Tittabawassee River floodplain and areas of the city of Midland, leading to an increase in residents’ body burdens of polychlorinated dibenzodioxins and polychlorinated dibenzofurans.

**Objectives:**

The UMDES is a hypothesis-driven study designed to answer important questions about human exposure to dioxins in the environment of Midland, where the Dow Chemical Company has operated for > 100 years, and in neighboring Saginaw, Michigan. In addition, the UMDES includes a referent population from an area of Michigan in which there are no unusual sources of dioxin exposure and from which inferences regarding the general Michigan population can be derived. A central goal of the study is to determine which factors explain variation in serum dioxin levels and to quantify how much variation each factor explains.

**Conclusions:**

In this article we describe the study design and methods for a large population-based study of dioxin contamination and its relationship to blood dioxin levels. The study collected questionnaire, blood, dust, and soil samples on 731 people. This study provides a foundation for understanding the exposure pathways by which dioxins in soils, sediments, fish and game, and homegrown produce lead to increased body burdens of these compounds.

The University of Michigan Dioxin Exposure Study (UMDES) was undertaken in response to concerns that the discharge of dioxin-like compounds from the Dow Chemical Company facilities in Midland, Michigan, resulted in contaminated soils in the Tittabawassee River floodplain and areas of the city of Midland, leading to an increase in residents’ body burdens of poly-chlorinated dibenzodioxins (PCDDs) and polychlorinated dibenzofurans (PCDFs). The Dow Chemical Company has operated in Midland since 1897 and is believed to have caused two major patterns of environmental contamination: *a*) an aerosol plume from historic incinerators that deposited PCDDs on surficial soils downwind of the plant, principally to the north and northeast in the city of Midland; and *b*) contamination of the Tittabawassee River downstream of the Dow plant to the southeast with materials from chloralkali operations dating back to the World War I era. In addition, this facility was a major producer of 2,4-dichlorophenoxy-acetic acid and 2,4,5-trichlorophenoxyacetic acid during the Vietnam conflict era and has produced a number of products derived from chlorophenols. The contaminant distribution of the Tittabawassee River is currently undergoing extensive mapping, and heavily contaminated areas are being remediated.

The goals of the study focus on assessing the human body burdens of dioxins [the 29 PCDD, PCDF, and polychlorinated biphenyl (PCB) congeners with dioxin-like activity (Van den Berg et al. 1998, 2006)] and the factors that predict those body burdens. Other instances of environmental exposures to dioxins have resulted in increased body burdens of these compounds: residents of Seveso, Italy, who were exposed by a release from a trichlorophenol reactor in 1976 (Bertazzi et al. 2001; Landi et al. 1998); the Ranch Hand cohort (Akhtar et al. 2004) and Vietnamese civilians (Baughman and Meselson 1973; Dwernychuk et al. 2002; Schecter et al. 1986, 2003) exposed to Agent Orange during the Vietnam era; and victims of the Yusho (Masuda 2003; Rappe et al. 1978) and Yucheng (Guo and Yu 2003) rice oil poisoning incidents in 1968 and 1979, respectively. These studies have documented the occurrence of chloracne among heavily exposed subjects and have suggested excess cancer incidence, diabetes, and other endocrine-related health effects. Thus, it is important to document exposure pathways and their relationship to the body burden of dioxins as a prerequisite to the determination of any potential health effects.

In this article, we describe the hypotheses to be tested; the design of the multistage population sampling; the survey instruments used; methods for collection of soil and household dust samples; the analytical methods for measuring dioxins in serum, household dust, and soil; the survey methods used to calculate sample weights; and the methods used for imputing item missing values.

The hypothesis to be tested is whether contamination of the environment by dioxins from the Dow Chemical Company’s operations in Midland, Michigan, is associated with increased body burdens of dioxins among some residents of the surrounding area of Midland, Saginaw, and southwestern Bay counties. For the purposes of this study, we use the term “contamination” to mean the presence of dioxins above background levels in environmental media, where “background” is defined as the concentration that would occur in an area without known point sources of that substance [U.S. Environmental Protection Agency (EPA) 2003]. The study includes populations who live both in and out of the Tittabawassee River floodplain and the plume area downwind of Dow, and who live in a region of Michigan (Jackson and Calhoun counties) that has no known industrial sources of dioxins. By studying these populations, it is possible to understand whether the serum dioxin levels among people who live in the Tittabawassee River floodplain are different than those among similar people who live in the same region of Michigan out of the floodplain and whether they are different than those among people who live in other parts of Michigan. An additional central goal of the study is to communicate the results and the implications of the results in an effective manner to the study participants and to the population in the Saginaw and Midland region.

A number of studies of PCDDs, PCDFs, and PCBs in human serum lipids (Ferriby et al. 2007; Lorber 2002; National Center for Environmental Health 2005; Patterson et al. 2004, 2008, 2009; Pinsky and Lorber 1998; U.S. EPA 2003; Wong et al. 2008) show that serum levels have a complex relationship with age, sex, race/ethnicity, birth cohort (and historic period of exposure), and congener half-life. There is a need for additional studies to examine the relationship between environmental media and human blood samples.

## Materials and Methods

### Schedule

We began developing the study protocol in the winter of 2004 and completed it in the summer of 2004. Field interviews, blood collection, soil sampling, and household dust collection were conducted in the summer and fall of 2004 and the spring and summer of 2005. All fieldwork ceased during the winter of 2005, when soil samples could not be collected because of frozen ground. Laboratory analyses of blood, soil, and household dust began as samples were collected and were completed in the spring of 2006. Statistical analyses of the data began in fall 2005.

### Sample design and subject selection

The sample design was a stratified, multistage area probability sample of households and persons. We defined the population as persons residing in Midland and Saginaw counties, Williams Township in Bay County, and Jackson and Calhoun counties, all of whom were > 18 years of age, had lived in their current residence continuously for at least 5 years, and currently lived in a residence outside the floodplains of the Shiawassee and Saginaw Rivers in Saginaw County. The sample used a two-stage area probability selection of housing units in the study area, and a third stage of selection of an eligible person within each sample housing unit. The first stage of selection employed stratified cluster sampling methods in which a sample was drawn from a list of all U.S. Census blocks in the study counties.

**Figure 1:**
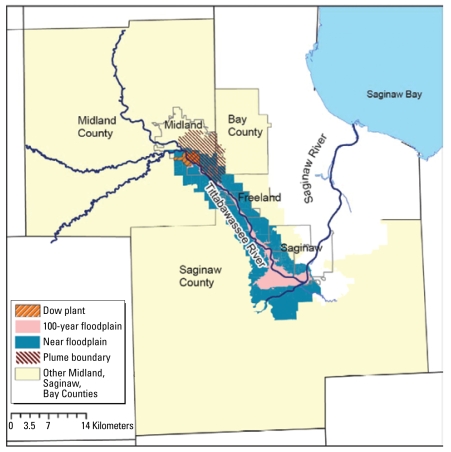
Map of Midland, Saginaw, and Bay counties, Michigan, showing the Dow Plant and the 100-year floodplain of the Tittabawassee River.

We divided the list into four groups (Figure 1): *a*) blocks in Midland and Saginaw and parts of Bay counties that contained any land area in the Federal Emergency Management Administration–defined 100-year floodplain of the Tittabawassee River below the Dow Chemical Company facility in Midland, and above the point where the Tittabawassee and Shiawassee Rivers join and have a mixed floodplain; *b*) blocks in the area of deposition from emissions stacks at the Dow Chemical Company in Midland, as defined by environmental modeling of the plume of the historical emission data; *c*) blocks outside of the Tittabawassee floodplain (item *a* above) or the plume (item *b* above) and outside the flood-plain of the Shiawassee and Saginaw rivers; and *d*) blocks in Jackson and Calhoun counties (control area for the study).

We defined the aerosol plume by a geostatistical simulation-based method that combined the process-based modeling of atmospheric deposition from an incinerator with the probabilistic modeling of residual variability of field samples. We used the approach to delineate areas with high levels of dioxin around the Dow plant, accounting for 53 field data points and the output of the U.S. EPA Industrial Source Complex (ISC3) dispersion model. We simulated 100 realizations of soil toxic equivalent (TEQ) values on a grid with a 50-m spacing. We used these realizations to identify census blocks (Figure 1) that were predicted to have elevated soil TEQ values (Goovaerts et al. 2008a, 2008b).

We recruited and hired interviewers from Midland and Saginaw counties. They were trained in general interviewing techniques, the specific study protocol and questionnaire, and refusal aversion techniques. Study staff monitored daily data collection progress, which achieved high response rates. Sample households were visited by interviewers multiple times, if necessary, to obtain cooperation. Interviewers also offered a financial incentive totaling $100 if the person participated in the interview and the blood, soil, and household dust sampling.

Each household was screened to determine whether eligible persons lived in the household. If one or more eligible persons lived in the household, we chose one at random to interview. Each respondent eligible for the blood draw was asked to provide a blood sample collected through an in-home visit from a phlebotomist from a local health care facility. If the respondent owned the housing unit, she/he was asked to permit household dust to be collected by vacuuming. We asked respondents who owned the property to permit soil samples to be gathered from around the housing unit (excluding apartments and condominiums).

In fall 2004, the first replicate in Midland and Saginaw counties was available for study. “Replicate” refers to random samples chosen from a target population, in which a first sample is taken and then a replicate sample is taken from the same target population. Replicate sampling is valuable in estimating the variances of parameter estimates. In spring 2005, the second replicate in Midland and Saginaw counties and the entire sample in Jackson and Calhoun (the control) counties were available for study.

Between the fall 2004 and spring 2005 data collection, we converted the survey interview from paper-and-pencil format to computer-assisted format on laptop computers. The paper-and-pencil and computer-assisted interviews both asked the same questions, were subjected to the same quality controls, and were of comparable quality. The questionnaire response rates varied between 82% and 84% in the different study populations (Table 1). Households and persons failing to respond to interview requests were recontacted, and those who cooperated answered a shorter questionnaire containing the same questions on the key study variables, with the same financial incentive, to determine whether substantial differences existed between respondents to the full survey and those who refused or could not be interviewed in the fall data collection. The spring data collection also achieved higher than expected response rates to the interview and the blood, dust, and soil collection. We followed the spring data collection with additional nonresponse interviewing with a shortened questionnaire.

A commonly used indicator of survey quality is the response rate. Cooperation rates were substantially higher than anticipated: 84% in the floodplain and 82% in the nonfloodplain and control areas. The overall interview response rate (74%) is lower than the cooperation rate because the response rate incorporates an estimate of the proportion of eligible persons in households that were not successfully screened. Table 1 shows cooperation rates at each stage, as well as the final interview response rate computed following guidelines from the American Association for Public Opinion Research (2006). We did not calculate response rates for the plume area separately because we included it in the nonfloodplain area. About 10% of the houses were not successfully screened because the household could not be contacted despite repeated attempts or because members of the household were not interested or did not have the time to provide a household listing.

Surveys often make adjustments to compensate for missing values, such as occurred in the UMDES. These models produced predicted probabilities of cooperation. The inverse of these predicted probabilities for respondents at each stage were then used as nonresponse adjustment factors and multiplied times the unequal probability of selection weight for each person. We “trimmed” extremely large values (i.e., reduced them to a smaller value) because the weighted value could be overly influential in an estimate. We then used these weights in all analyses to compute weighted estimates that would be sound estimates for the population from which the sample was drawn (Kish 1965). For example, for the TEQ blood value in parts per trillion for the *i*th person, *y_i_*, and nonresponse adjusted weight, *w_i_*, we computed the weighted mean TEQ value for the population as


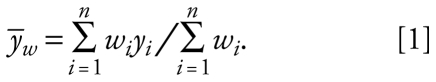


We used the same “global” weight for all analyses.

We further adjusted the data to account for missing values for items or missing values for a single variable for an individual who otherwise provided data. Many analysts “ignore” the missing values in a variable by using “case-wise deletion” of missing data features in statistical software. Ignoring the missing values effectively imputes or assigns the mean of the cases without missing values to the value for each case for which the value is missing. In the careful population inference methods being used for the UMDES, we imputed the item missing values in the survey questionnaire, the household dust, and the soil samples. We used a sequential regression imputation procedure (Raghunathan et al. 2001) to replace values for missing items in the UMDES questionnaire data, and used imputed values in estimating various statistics from the survey.

### Nonresponse follow-up study

For a subset of households that were not contacted or for which eligibility was not determined, or for which the selected respondent did not complete the questionnaire, we asked them again to participate in the UMDES. A shortened questionnaire, which collected information on key study variables, was administered to subjects who agreed to participate in this non-response follow-up study. We did not collect blood, soil, and household dust samples. These data permitted comparisons of nonrespondents and respondents on key study variables from the questionnaire to assess the degree of non-response bias. Nonresponse follow-up activities took place during January and February 2005 and October and November 2005, immediately after the end of main data collection. We constructed nonresponse adjustment weights using response propensity models, which use a logistic regression predicting the likelihood of being a respondent, conditional on being eligible for response. Models were run for each of the following stages of nonresponse: *a*) contact, given status as an occupied housing unit; *b*) screening, given initial contact; *c*) interview, given successful screening and eligibility for the interview; *d*) giving blood or not, conditional on being eligible to give blood; *e*) giving dust, conditional on being eligible to give dust; *f*) giving soil, conditional on being eligible to give soil; and *g*) giving blood, dust, and soil, conditional on being eligible to give all samples.

We used the inverse of predicted probabilities from each of these models as a non-response adjustment weight. We trimmed extremely small predicted probabilities from each of these models to minimize the influence of any single case on the overall estimation, while maintaining as much of the original predictions as possible.

### Interviews

Survey questionnaires were developed through a process of writing or adopting questions from other surveys, review by project stakeholders and the scientific advisory board (SAB), and pretesting in a small sample of residents in Midland and Saginaw counties. All eligible adults gave written informed consent and completed a 1-hr standardized interview administered by the Survey Research Center at the University of Michigan.

An important component of the questionnaire was an event history calendar (EHC), which collects significant time-varying information using cues from the respondent’s lifetime to assist in recall. The interviewer recorded major life events, such as marriage or childbirth, on the EHC, along with major national and local current events to help respondents anchor when events occurred. The questionnaire consisted of 10 sections, each of which contained lifetime recall questions. The respondent was asked to recall possible dioxin exposure pathways over their entire lifetime in 1-year intervals. Much of the interview was devoted to questions about consumption of fish, game, poultry, dairy, and produce and whether it came from contaminated areas; activities such as hiking, camping, picnicking, and water sports in the contaminated areas; occupational history, particularly work at Dow and in other settings where PCDD, PCDF, or PCB exposure would have been likely; and residential history. All questions included the historic periods when the activities occurred. The complete interview questionnaire is available on the UMDES website (University of Michigan 2008).

### Confidentiality procedures and protection of human subjects in research

The study was unusual in that it addressed the potential economic risks to subjects from participation. The Michigan Department of Environmental Quality considers any property with soil levels ≥ 90 ppt TEQ for dioxins, furans, and PCBs to be a “facility” for the purposes of state regulations (Michigan Department of Environmental Quality 2001, 2005). Thus, participation in the UMDES potentially carried risks of financial harm to participants from disclosure of this data. Moreover, if a subject simply gained knowledge of levels of dioxins, furans, and PCBs in his or her soil and/or household dust, it could have negative consequences on the value of a subject’s home and/or property. Sensitive and personally identifiable information concerning participants included responses to interview questions and concentrations of dioxins, furans, and PCBs in their blood, household dust, and soil. To protect the confidentiality of participants and their data, we obtained a Certificate of Confidentiality from the National Institutes of Health (Bethesda, MD), which protects the data in perpetuity against the compelled disclosure of personally identifiable information.

Although there are no state regulations regarding the dioxin content of household dust (as there are for soil), and there are no medical guidelines for interpreting serum dioxin levels, participants were given the option to receive or not receive their results for each of their samples (blood, dust, and soil). This allowed participants to protect themselves from potential economic damages consequent to participation and awareness that their property was contaminated. Essentially, participants who did not know their soil and household dust results would be identical (with respect to economic risk) to subjects who were not selected for study or who chose not to participate after being selected. The text of all informed consent forms and written communications with potential subjects are available on the UMDES website (University of Michigan 2008). All staff and contractors who participated in the field operations, data collection, and data management, in addition to members of the SAB, were required to sign confidentiality agreements that stipulated the procedures that would be followed to protect the data and the staff member’s agreement to protect the data from release. Each participant was assigned a unique identification code, and data were identified only by the numeric code. All aspects of the study were approved by the University of Michigan institutional review board.

### Blood collection and analysis

During the interview, each subject was asked questions that established eligibility for blood drawing [based on the Red Cross criteria for blood donors (e.g., no clotting disorders or blood thinner medications, no recent chemo therapy, and weight of at least 110 lb)]. Each eligible participant was asked to give an 80-mL sample of blood. Blood was usually drawn in the participant’s home by a mobile phlebotomy service in each community and was delivered on wet ice to the laboratory of either Mid-Michigan Medical Center (for Midland, Saginaw, and Bay counties) or Foote Hospital (for Jackson and Calhoun counties).

In the laboratory, blood was allowed to clot and then was centrifuged, and the serum was decanted. Serum was frozen at −20°C and was shipped by express mail on dry ice to Vista Analytical Laboratory (El Dorado Hills, CA). Vista performed analyses for the 29 dioxins, furans, and PCBs for which consensus toxic equivalency factors (TEFs) have been published (Van den Berg et al. 1998, 2006), using modified U.S. EPA protocol 8290 (U.S. EPA 1994) and 1668 (U.S. EPA 1999) for sample extraction and quantitation. Approximately 25 mL of the collected serum sample was used for the analysis; samples were spiked with ^13^C_12_-labeled internal standards, and the analytes of interest were extracted with hexane and concentrated. The extracts were then fractionated using silica gel and activated alumina columns. An aliquot of each extract was injected into a gas chromatograph and the selected analytes were quantified by high-resolution mass spectrometry on a Waters Ultima magnetic-sector high-resolution mass spectrometer (Waters Corporation, Milford, MA) using selected ion monitoring at 10,000 resolving power. The concentration of each analyte was then calculated based on a standard linear calibration specific to each congener. Each analytical run, which was blinded to the analyst, consisted of the unknown serum samples, a method blank (quality control), an ongoing precision and recovery sample (quality control), two solvent blanks, and two calibration standard solutions. After all quality assurance and quality control data were reviewed, the analytical results were calculated on both a whole-weight and a lipid-adjusted basis. Serum total lipids for each sample were calculated using Phillips formula summing triglycerides and total cholesterol.

Table 2 lists dioxin congeners and median limits of detection (LOD) for whole-weight and lipid-adjusted–weight congeners. A total of 946 persons had analyzable blood samples (Table 3).

### Household dust sampling and analysis

Dust sampling was conducted in the home of each respondent who had completed the interview and blood draw, after consent of the respondent, if the respondent was an owner of the residence. Participants who did not own the residence (e.g., renters, adult children of owners) did not have legal authority to give permission for sampling without the property owner’s consent. In consequence, their dust was not sampled, even though they otherwise participated in the study. All household dust samples were collected using high-volume small-surface samplers (HVS3; CS-3, Inc., Sandpoint, ID). Vacuums were equipped with a cyclone and a fine-particle filter capable of capturing 99.95% of particles > 0.3 μm in mean aerodynamic diameter. One composite sample was collected in each household from sampling locations that presented the highest potential for human contact with dust. Locations were generally a frequently occupied living space (living room or family room) and a high-traffic pathway (front hall, kitchen entryway, or other high traffic hallway). Samples were taken from both hard and soft surfaces, with carpets and area rugs being preferred sampling surfaces. Samples were not taken from undisturbed dust in generally inaccessible areas because there was no way to tell when such samples became contaminated or whether there had been any exposure by the participants. The sampling protocol was based, with minor modifications, on the American Society for Testing and Materials (ASTM) D 5438-00 method (ASTM 2000). The sampling technicians attempted to collect a minimum of 10 g total dust.

The sampling technician recorded the total surface area of the sampling area (typically 1–2 m^2^) on a preprinted field data sheet, as well as the surface types where the sample was taken. Technicians transported samples on ice in a dedicated 4°C cooler until delivery to Vista Analytical Laboratory for analysis of the World Health Organization (WHO) 29 PCDD, PCDF, and PCB congeners using internal modifications of U.S. EPA method 8290 (U.S. EPA 1994) and method 1668 (U.S. EPA 1999), and using the laboratory methods described above for serum samples. LODs were between 1 and 8 pg/g for all PCDD, PCDF, and PCB congeners except octachlorinated dibenzofurans (OctaCDF), PCB-114, PCB-123, and PCB-167, for which the LODs were between 20 and 40 pg/g (Table 2). For household dust dioxin concentrations < LOD, we assigned a value equal to the LOD divided by the square root of 2 ( 
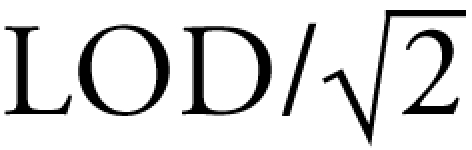
). We sampled a total of 764 residences for household dust (Table 3).

### Soil sampling and analysis

**Figure 2:**
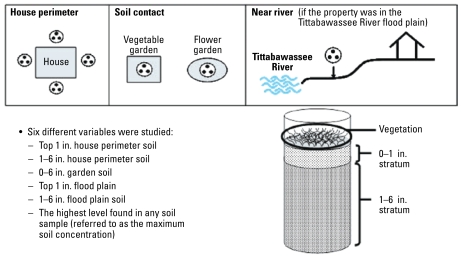
Schematic location of soil samples from each participants’ property and stratification of soil samples indicating three soil samples taken within each ring.

Soil sampling was conducted at the residence of each respondent who had completed the interview and blood draw after giving consent, if the respondent was an owner of the residence. Participants who did not own the property (e.g., renters, condominium owners, and adult children of owners) did not have legal authority to give permission for sampling without the property owner’s consent. In consequence, their soil was not sampled, even though they otherwise participated in the study. Each property was sampled in multiple locations by a sampling technician using a push core sampler to collect a core of soil from the surface to a depth of 6 in. (15 cm). Surface vegetation at the site of the core was also collected except in situations where garden plants might be damaged. For selection of locations for sampling, technicians followed a protocol that identified the house perimeter, property areas where skin contact was likely (e.g., gardens), and areas in or near the floodplain of the Tittabawassee River. The sampling protocol is portrayed schematically in Figure 2.

Up to four sampling stations were located around the perimeter of the house (areas covered by pavement, cement, or gravel were not sampled). We determined locations where activities occurred that were likely to result in skin contact with soil from the interview responses. If the respondent worked in a flower garden, it was sampled. If there was a vegetable garden, it was sampled regardless of whether the participant worked in it, based on the assumption that the homegrown vegetables were consumed by all members of the household. Up to two gardens (soil contact areas) were sampled. For residences located in the floodplain, one additional station at the lowest, safely accessible location on the respondent’s property in the floodplain was sampled, referred to as the near-river sample. Thus, each residence had a maximum of seven sampling stations (four house perimeter samples, two soil contact samples, and one near-river sample).

Each sampling station was defined by laying out a 3-foot-diameter sampling ring, and three equally spaced cores were collected within the ring. All sample location coordinates were established using global positioning system procedures, for mapping purposes, and to relocate sample sites, if necessary. Technicians stored all sealed sample cores on wet ice (4°C) before transport to the University of Michigan Environmental and Water Resources Engineering laboratories, where they were extruded, separated into strata by depth, and composited across cores. Samples were stratified so that the top 0–1 in. (0–2.5 cm) could be examined separately from the 1–6 in. (2.5–15 cm) sample because surficial deposition of aerosols would be expected to affect only the top 1–2 cm of soil, whereas contamination from other pathways (e.g., fluvial deposition in the river floodplain) would be expected to affect deeper soil. Ultimately, each residence yielded the following analytical samples: house perimeter set 0–1-in. (0–2.5 cm) depth composite; house perimeter set 1–6 in. (2.5–15 cm) depth composite; and house perimeter set surface vegetation composite.

If there was a soil contact set, the residence yielded two additional samples: *a*) soil contact set 0–6 in. (0–15 cm) composite [we did not sample the top 1 in. (2.5 cm) separately because garden soil is routinely turned over, soil additives and mulch are routinely incorporated into the soil, and spring planting and pulling of plants in the fall routinely disturb the soil strata. Moreover, root vegetables such as carrots do not grow just in the top 1 in.]; and *b*)soil contact set surface vegetation composite (if available). In addition, residences in the Tittabawassee River floodplain yielded the following samples: near-river set 0–1 in. (0–2.5 cm) depth composite; near-river set 1–6 in. (2.5–15 cm) depth composite; and near-river set surface vegetation composite.

A total of 766 residences were sampled in the five counties in Michigan from October through December 2004 and from March through September 2005. A total of 2,081 samples were analyzed for the 29 dioxin, furan, and PCB congeners (Table 2) by Vista Analytical Laboratory using internal modifications of U.S. EPA methods 8290 (U.S. EPA 1994) and 1668 (U.S. EPA 1999) as described above for serum samples. LODs were substantially < 1 pg/g for all PCDD, PCDF, and PCB congeners except PCB-167 (LOD = 3 pg/g; Table 2). We assigned soil dioxin concentrations that were less than the LOD a value of 
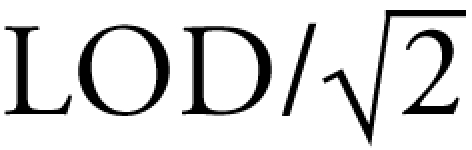
.

All of the 0–1 in. (0–2.5 cm) house perimeter composite samples were analyzed. If any part of the property was in the floodplain, then we also analyzed all remaining composites (1–6 in. and vegetation house perimeter; 0–1 in., 1–6 in., and vegetation floodplain; and 0–6 in. and vegetation soil contact). If the respondent did not live in the floodplain but had a vegetable garden or worked in a flower garden, we analyzed the 0–6 in. and vegetation composites for the soil contact set. If the TEQ of the 0–1 in. house perimeter composite for any property outside the floodplain was > 8 pg/g, then we analyzed the 1–6 in. and vegetation house perimeter composites. The trigger value of 8 pg/g TEQ represents the 75th percentile of the background distribution for the lower peninsula of Michigan (i.e., we expected 25% of soil samples to be > 8 pg/g).

### Stakeholder involvement

Stakeholders, which we defined as entities that had a direct interest and that were actively involved in the dioxin issue in Midland/Saginaw [including the Michigan Department of Community Health; Michigan Department of Environmental Quality; Midland, Saginaw, and Bay county health departments; environmental groups (Lone Tree Council and Ecology Center); the Dow Chemical Company; and the Agency for Toxic Substances and Disease Registry (ATSDR)] played a key role in the development of the study protocol. We held numerous face-to-face and telephone meetings with stakeholders to review the study goals, objectives, and draft protocol. Stakeholders submitted multiple sets of written comments, and the study team provided written responses that were posted on the UMDES website (University of Michigan 2008). The process of stakeholder involvement resulted in major modifications to the study protocol, including: *a*) important changes to the questionnaire; *b*) addition of the Midland plume area as a separate study group; and *c*) addition of Jackson and Calhoun counties as control areas. Stakeholders did not participate in the selection of the study participants, sample collection, or laboratory or statistical analyses. Stakeholders were invited to all SAB meetings, and they had opportunities to discuss the study design, conduct, and analysis with the investigators and privately with the SAB. We treated the Dow Chemical Company in the same manner as all other stakeholders.

### The SAB

We reported to an independent SAB made up of four scientists who were nominated by the stakeholders and appointed by the University of Michigan, with membership based on independence, qualifications in research relevant to dioxin issues, and scientific stature. Neither Dow nor any other stakeholders played any role in the selection of the SAB members. The members of the SAB are listed in the acknowledgments; their affiliations are posted on the UMDES website (University of Michigan 2008). The SAB oversaw all aspects of the study conduct, including *a*) reviewing and commenting on the draft study design; *b*) convening in person in Michigan twice yearly for 1–2 days each time to meet with the investigators, representatives of the Michigan Department of Community Health and other health officials, representatives of the community advisory panel (CAP), and stakeholders; *c*) monitoring the conduct of the UMDES; *d*) providing feedback to the investigators regarding the conduct of the UMDES; and *e*) reviewing and commenting on draft reports and manuscripts before they were released to the public and scientific community.

### Role of the Dow Chemical Company

Funding for this research was available through an unrestricted grant from the Dow Chemical Company to the University of Michigan. We gave Dow periodic accounting reports to assure Dow that funds were properly spent on study activities. We reported research progress and results to Dow only in public settings at the SAB–stakeholder meetings, open scientific conferences, and meetings with county, state, and federal agencies and in public meetings in the Midland/Saginaw area. Dow played no role in the study design, data collection, data analysis, or data interpretation beyond providing written comments that the investigators posted to the UMDES website (University of Michigan 2008).

### Communications plan and CAPs

Because potential exposures to environmental toxicants such as dioxins are a public health concern, residents and public health professionals in the Tittabawassee River area had a great interest in the design and execution of this study. The research team was committed to proactive community engagement in the design and implementation of the study. The research team’s community outreach efforts had three key areas:

- We conducted research and focus groups to clarify the concerns of the community. We identified key community leaders, including elected officials, school superintendents, clergy, members of the news media, and heads of nonprofit organizations, whom we selected for interviews. These investigations allowed identification of areas that could be addressed by the study team and helped to guide interactions with the community.
- We formed two CAPs (one for Midland/Saginaw/Bay counties and one for Jackson/Calhoun counties) with membership based on independence, representation of community groups, and stature and respect in the community. We solicited nominees during focus groups and key-person interviews. The CAPs provided feedback to us regarding the concerns of the community, and they helped to inform the community about the conduct and progress of the study. The memberships of the CAPs are posted on the UMDES website (University of Michigan 2008).
- We developed a broad outreach/educational campaign to describe the efforts of the research team and to provide critical information to the public. The campaign involved media resources at the University of Michigan and in the communities, website development, area physicians, elected officials, public health officials, key community leaders, and regular, open meetings with the public. The outreach/educational campaign included descriptions of the research study, periodic updates on study progress, findings from the study as they became available for release, and interpretations of the findings [examples are available on the UMDES website (University of Michigan 2008)]. We conducted research to determine how best to communicate results that are relevant to the community’s needs and concerns. Individual participants were sent the results of their tests (measurements of serum, household dust, and soil) by mail, if they wished to receive them. We disseminated a 41-page booklet with a lay summary of results to the study participants and to the general public. Aggregate data are being presented in scientific reports that are posted to the UMDES website (University of Michigan 2008) after peer-review by the SAB.

## Results and Discussion

This study has a number of unique features. First, informed consent for taking soil and household dust samples included discussion of the potential economic risks to subjects from participation in the research. Recent publications have highlighted the importance of this issue (Brody et al. 2007). This is a complex issue that other researchers need to consider and that we discussed at length with our institutional review board and with legal counsel for the University of Michigan. An adult may give consent to be interviewed and provide a blood sample without consideration of other parties’ rights. However, taking household dust and soil samples must include consideration of who actually owns the sample. If a subject lives in a condominium, for example, the subject owns the structure but does not own the land on which the building is located and therefore does not have an unabridged right to give a soil sample. The consent of the property owner should also be obtained, particularly where the value of the property may be adversely affected by finding contaminants in the soil. Obtaining consent of the property owner would have revealed that the subject was a participant in the study, which would necessarily have violated the confidentiality of the participant; for this reason we did not perform soil sampling unless the participant owned the land.

We considered household dust to be the property of the participant, provided that the participant owned the structure (e.g., a house or condominium), whether or not the participant owned the land. In these instances, we asked the participant to consent to household dust sampling. In instances where the participant was the adult child (or other relative) of and lived with the property owner, we did not take soil or dust samples. Again, this would have violated the confidentiality of the participant.

Almost all of the participants (> 98%) asked to receive the results of their blood dioxin analyses. In contrast, only 64% asked to receive their soil results. We believe this lower number reflected the potential economic risks to participants from knowing the contamination levels on their properties. Protection of the study participants included protection from economic risk consequent to their knowing the dust and soil results from their property. By not receiving their results, they could participate in the study yet be no different in terms of risk than nonparticipants.

Our participation rates were high, even in the control areas (Jackson/Calhoun counties); in fact, these rates were higher than we anticipated when we planned the study. We believe this was due to the intense concern in the contaminated area regarding the risks of dioxin pollution and the widespread desire of the general public to participate in characterizing these risks and in taking appropriate actions to reduce risks. Communications with the affected population were a central part of this study and have been ongoing since the study planning began. Including the affected population in the study design, keeping them updated on study progress, and reporting the results in public meetings in a timely manner have led to widespread acceptance of the findings as providing a factual basis for addressing the dioxin pollution.

We based our laboratory analyses on large samples of serum (~ 25 mL), household dust (10 g), and soil (10 g), which allowed us to achieve low LODs for all PCDD, PCDF, and PCB congeners. For the PCDD and PCDF congeners of greatest concern, we achieved low LODs, and few samples had unmeasured levels. This allowed us to make important inferences about the dioxin levels in the blood in the referent population, as well as to characterize the full range of the distribution for each congener in blood, household dust, and soil. Comparisons of mean and median values across populations, and inferences about differences in these measures between population groups, are greatly improved when they are based on measurements rather than assumptions about values < LOD. Our LODs for serum were substantially less than those reported for the National Health and Nutrition Examination Survey (NHANES) 2001–2002 data (Patterson et al. 2008) primarily because we had larger serum samples for analysis that comparable to those reported for the NHANES 2003–2004 data for most congeners (Patterson et al. 2009).

In this study we collected complete questionnaire, blood, dust, and soil samples on 731 people (Table 3) of the 1,324 who completed the questionnaire and the 946 who gave blood samples. We are not aware of any population-based studies that have included this large a sample of participants with concurrent blood, household dust, and soil measurements of dioxins and interview data about past exposures and activities in the contaminated area. Although we did not include measurements of the dioxin content of fish, game, or produce from the contaminated areas, we are conducting other research that will characterize these samples. Moreover, questionnaire information about past consumption of these foods is essential in determining whether they are associated with increased serum levels. The insights to be gained from this study will provide a strong foundation for understanding the exposure pathways by which dioxins in soils, sediments, fish and game, and homegrown produce lead to increased body burdens of these compounds, especially in settings where there has been extensive and prolonged environmental contamination.

An important concern is often raised in the investigation of industrial contamination sites, as illustrated by this study. On the one hand, high-quality, expensive research is needed to identify the extent of the contamination and the impact on the human population, which should be paid for by the entities that created the pollution. On the other hand, there is often concern in the affected communities that research paid for by the industry will not fairly address the issues. In the present instance, there are also concerns in the affected communities that neither the government agencies nor the environmental groups will fairly address the issues. We set up our study to provide a credible method by which community concerns could be addressed, and to that end we sought extensive community input and participation, rigorously protected the confidentiality of our participants, set up an independent advisory board to which we reported, had no reporting relationship to Dow, kept our methods and results transparent, and built an extensive communications program. These methods have resolved a myriad of practical problems and are applicable across a broad range of settings.

## Figures and Tables

**Table 1:** Cooperation rates for interview, blood, household dust, and soil sampling and final interview response rate, by study area.

Study area	Cooperation rate (%)				Interview response rate (%)
	Interview	Blood	Dust	Soil	
Floodplain/near floodplain	83.7	83.9	91.0	91.3	—
Other Midland/Saginaw and plume	82.4	73.7	90.9	93.2	—
Jackson/Calhoun	82.2	78.4	93.8	91.9	—
Total	82.9	79.6	91.7	92.0	74.3

**Table 2:** WHO TEFs (1998 and 2005) for humans and UMDES median LODs for blood, dust, and soil samples.

	TEF		LOD		
Congener	1998	2005	Serum, lipid adjusted (pg/g-lipid)^a^	Soil^b^ (pg/g)	Dust (pg/g)
PCDDs					

2,3,7,8-TCDD	1	1	0.5	0.2	0.5
1,2,3,7,8-PentaCDD	1	1	2.1	0.2	1.2
1,2,3,4,7,8-HexaCDD	0.1	0.1	2.6	0.4	2.2
1,2,3,6,7,8-HexaCDD	0.1	0.1	3.4	0.3	4.8
1,2,3,7,8,9-HexaCDD	0.1	0.1	2.6	0.5	2.6
1,2,3,4,6,7,8-HeptaCDD	0.01	0.01	2.1	0.3^c^	2.5
OctaCDD	0.0001	0.0003	2.4^c^	0.4^c^	2.2^c^

PCDFs					

2,3,7,8-TetraCDF	0.1	0.1	0.4	0.3	0.8
1,2,3,7,8-PentaCDF	0.05	0.03	0.4	0.2	1.0
2,3,4,7,8-PentaCDF	0.5	0.3	1.5	0.2	1.2
1,2,3,4,7,8-HexaCDF	0.1	0.1	2.2	0.2	1.0
1,2,3,6,7,8-HexaCDF	0.1	0.1	1.8	0.2	0.9
1,2,3,7,8,9-HexaCDF	0.1	0.1	1.1	0.1	0.7
2,3,4,6,7,8-HexaCDF	0.1	0.1	1.0	0.2	1.4
1,2,3,4,6,7,8-HeptaCDF	0.01	0.01	1.9	0.1^c^	5.8
1,2,3,4,7,8,9-HeptaCDF	0.01	0.01	1.0	0.2	1.5
OctaCDF	0.0001	0.0003	2.5	0.2^c^	20.8

PCBs					

3,4,4′,5-TetraCB (PCB-81)	0.0001	0.0003	1.4	0.2	2.8
3,3′,4,4′-TetraCB (PCB-77)	0.0001	0.0001	1.0	0.1^c^	0.4^c^
3,3′,4,4′,5-PentaCB (PCB-126)	0.1	0.1	4.7	0.4	8.1
3,3′,4,4′,5,5′-HexaCB (PCB-169)	0.01	0.03	3.7	0.4	1.2
2,3,3′,4,4′-PentaCB (PCB-105)	0.0001	0.00003	0^d^	0.8^c^	2.1
2,3,4,4′,5-PentaCB (PCB-114)	0.0005	0.00003	0^d^	0.6	38.6
2,3′,4,4′,5-PentaCB (PCB-118)	0.0001	0.00003	0^d^	0.7^c^	5.2^c^
2′,3,4,4′,5-PentaCB (PCB-123)	0.0001	0.00003	34.4	0.6	27.3
2,3,3′,4,4′,5-HexaCB (PCB-156)	0.0005	0.00003	0^d^	0.2	5.3
2,3,3′,4,4′,5′-HexaCB (PCB-157)	0.0005	0.00003	0^d^	0.5	5.2
2,3′,4,4′,5,5′-HexaCB (PCB-167)	0.00001	0.00003	0^d^	3.0	31.6
2,3,3′,4,4′,5,5′-HeptaCB (PCB-189)	0.0001	0.00003	0^d^	0.3	1.8

a Data are pg/g-lipid, equivalent to or parts dioxin per trillion parts lipid.

b Soil samples from the house perimeter top 1 in. (0–2.5 cm).

c Median LOD among all samples (none were < LOD).

d LOD < 0.0005 pg/g.

**Table 3:** Number of participants, by region.

Sample type	Floodplain	Near floodplain	Midland plume	Other Midland/Saginaw	Jackson/Calhoun	Total across all areas
Interviews	326	264	71	304	359	1,324
Blood	251	197	48	199	251	946
Household dust	207	159	37	163	198	764
Soil	203	164	37	168	194	766
Interviews, blood, dust, and soil	195	156	35	162	183	731
